# False-Negative Results of Endoscopic Biopsy in the Diagnosis of Gastrointestinal Kaposi's Sarcoma in HIV-Infected Patients

**DOI:** 10.1155/2012/854146

**Published:** 2012-11-26

**Authors:** Naoyoshi Nagata, Katsunori Sekine, Toru Igari, Yohei Hamada, Hirohisa Yazaki, Norio Ohmagari, Junichi Akiyama, Takuro Shimbo, Katsuji Teruya, Shinichi Oka, Naomi Uemura

**Affiliations:** ^1^Department of Gastroenterology, Research Institute, National Center for Global Health and Medicine, 1-21-1 Toyama, Shinjuku-ku, Tokyo 162-8655, Japan; ^2^Department of Clinical Pathology, Research Institute, National Center for Global Health and Medicine, Tokyo, Japan; ^3^AIDS Clinical Center (ACC), Research Institute, National Center for Global Health and Medicine, Tokyo, Japan; ^4^Department of Infectious Disease, Research Institute, National Center for Global Health and Medicine, Tokyo, Japan; ^5^Department of Clinical Research and Informatics, International Clinical Research Center, Research Institute, National Center for Global Health and Medicine, Tokyo, Japan; ^6^Department of Gastroenterology and Hepatology, Kohnodai Hospital, National Center for Global Health and Medicine, Chiba, Japan

## Abstract

Kaposi's sarcoma (KS) is a rare endothelial neoplasm mainly involving the skin, but it is often associated with AIDS. Diagnosis of gastrointestinal (GI) tract KS, a common site of visceral involvement in AIDS, is important, but endoscopic biopsy carries a risk of false-negative results (FNRs) due to its submucosal appearance. This study sought to determine the rate and causes of FNR for endoscopic biopsy of GI-KS lesions. Endoscopic biopsy samples of 116 GI-KS lesions were reviewed retrospectively. All GI-KS lesions were confirmed to be resolved following KS therapy. FNRs were yielded for 41 of the lesions (35.3%). Among upper and lower GI sites, the esophagus was the only site significantly associated with FNRs (*P* < 0.01). Small size (<10 mm) and patches found on endoscopy were significantly associated with FNRs (*P* < 0.05). Findings of submucosal tumor (SMT) with ulceration were significantly associated with true-positive results (*P* < 0.05). In conclusion, FNRs were found in 35.3% of GI-KS lesions and were especially related to the site of the esophagus and endoscopic early stage (small size or patch appearance). An SMT with ulceration may be relatively easy to diagnose on endoscopic biopsy. Caution should be exercised when performing endoscopic biopsy of these lesions in AIDS patients and evaluating the histological features.

## 1. Introduction

Kaposi's sarcoma (KS) is a cancer of the lymphatic and blood vessels that mainly involves the skin [1–3]. It is a rare cancer but has become more widely known as one of the AIDS-defining illnesses [2, 3]. Although the rate of AIDS-related KS has decreased dramatically since the introduction of highly active antiretroviral therapy (HAART) [4–6], KS remains the most common malignancy among patients with AIDS [7].

KS can also involve the oral cavity, lymph nodes, and viscera [1–3, 8]. The diagnosis of visceral KS is important because the need for treatment and choosing among the various options depend upon the extent of disease [8–10]. The gastrointestinal (GI) tract is a common site of visceral involvement [11–15], and a definitive diagnosis of GI-KS can be made by endoscopic tissue biopsy [8, 16, 17]. Histopathologically, GI-KS is characterized by spindle cells that form vascular channels, which fill with blood cells [17, 18]. Endoscopically, GI-KS has various macroscopic presentations: patches, polypoid lesions, submucosal nodules, bulky masses, and ulcerations [13, 17, 19–23]. For submucosal nodules especially, endoscopic biopsy sampling has been known to yield false-negative results (FNRs) [17, 23–25]. Some GI-KS lesions might be more difficult to identify histologically depending on their location, size, or macroscopic appearance; however, which findings are related with false-negative histological results remain unknown.

The purpose of this study was to determine the rate and causes of FNR from endoscopic biopsies of GI-KS lesions. 

## 2. Materials and Methods

### 2.1. Subjects

Histopathology slides of endoscopic biopsy samples of 116 consecutive, GI-KS lesions from 24 HIV-infected patients who had not received anti-KS therapy were retrospectively reviewed. All biopsies were performed between 2002 and 2006 at the National Center for Global Health and Medicine (NCGM), a 900-bed hospital located in the Tokyo metropolitan area with the largest referral center for HIV/AIDS in Japan. 

### 2.2. Ethics Statement

The institutional review board at NCGM approved this study. All patients from whom clinical samples were obtained during endoscopic biopsy provided written informed consent prior to the procedure. Data obtained from the patient medical records was anonymized before analysis.

### 2.3. Clinical Factors

HIV infection route was determined by the medical staff who interviewed each patient on the first visit to our hospital. Routes of HIV infection were determined by medical staff who questioned each patient face to face on the first visit to our hospital. Routes were classified into six categories: homosexual, bisexual, heterosexual, drug user, untreated blood products, and unknown. Patients who were homosexual or bisexual were regarded as men who have sex with men (MSM).

CD4^+^ cell counts were checked within 1 week of endoscopy. HIV-RNA viral loads (VLs) determined by real-time quantitative polymerase chain reaction (PCR) were reviewed within 1 month of endoscopy. The minimum detection level was 40 copies/mL of plasma. A positive result for real-time HIV-RNA was defined as ≥40 copies/mL. 

### 2.4. Diagnosis of GI-KS

Biopsy was performed using biopsy forceps (FB-240U, FB230-K; Olympus Co., Tokyo, Japan). All biopsies were performed by well-trained endoscopists (experience of >1.000 colonoscopies). 

A definitive diagnosis of GI-KS was defined as follows.

**Figure 1:**
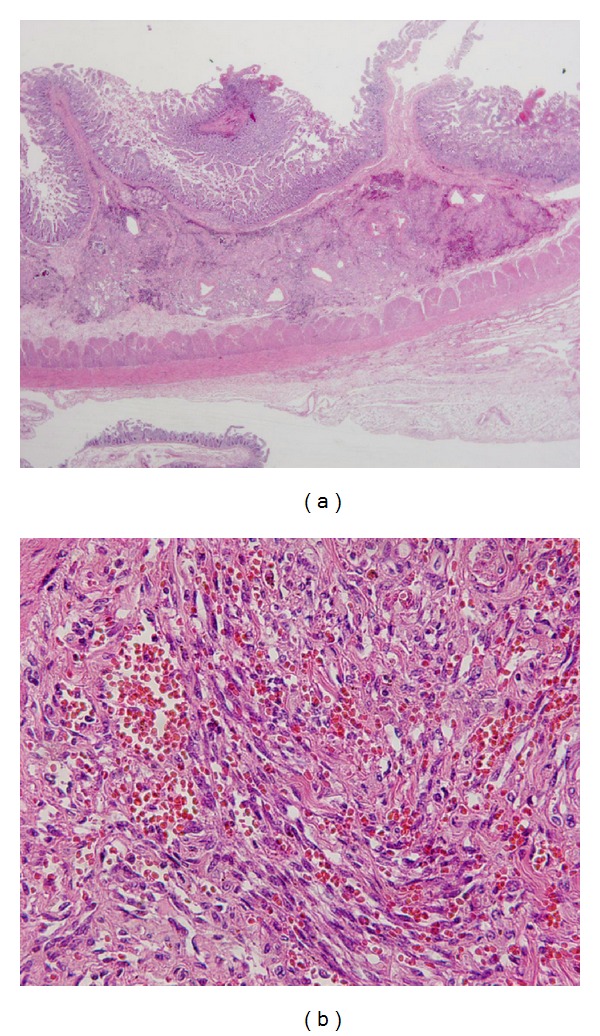
Pathological features of GI-KS on HE staining. (a) Low-power view showing a distinct proliferative lesion within the submucosa of the small bowel intestine. (b) High-power view showing spindle cell proliferation with vascular channel formations filled with blood cells.

1. Negative results confirmed from biopsy samples for other GI diseases such as infection, inflammatory bowel disease, hyperplastic polyp, fundic gland polyp, inflammatory polyp, adenomatous polyp, angioectasia, GI lymphoma, premalignant lesion, esophageal cancer, gastric cancer, and colorectal cancer.
2. Presence of proliferating spindle cells (Figure 1) with vascular channel formations filled with blood cells (Figure 1) seen on hematoxylin and eosin (HE) staining. Lesions with the absence of these findings from biopsy samples were defined as FNR.
3. Positive response to KS therapy (HAART or systemic therapy of liposomal anthracycline); in particular, for visible GI-KS lesions without typical pathological findings from biopsy specimens, partial or complete resolution confirmed on follow-up endoscopy following KS therapy.

### 2.5. Endoscopic Assessment of GI-KS

**Figure 2:**
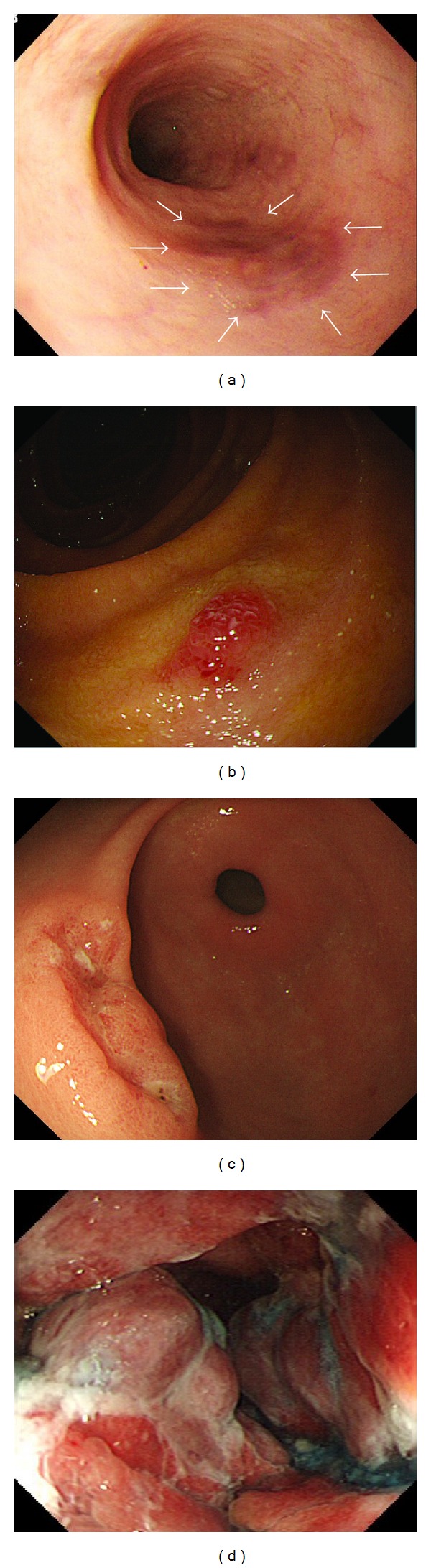
Gastrointestinal Kaposi's sarcoma on endoscopy. (a) Dark reddish patch lesion (arrow) in the esophagus. (b) Small (≤10 mm) and polypoid lesion in the duodenum. (c) Submucosal tumor-like lesion with ulceration in the stomach. (d) Bulky tumor mass surrounding the anorectal area causing anorectal stenosis.

GI-KS was evaluated in terms of site, size (≤10 mm or >10 mm), and macroscopic findings on endoscopy. Site of GI involvement was classified into 7 regions: esophagus, stomach, duodenum, ileum, right-side colon (cecum, ascending colon, and transverse colon), left-side colon (descending colon and sigmoid colon), and rectum. Macroscopic findings were evaluated in the presence of reddish mucosa with patches (Figure 2(a)), polypoid lesion (Figure 2(b)), submucosal tumor (SMT), SMT with ulceration (Figure 2(c)), and bulky mass (Figure 2(d)), as previously reported [13, 17, 19–23]. Ulceration was defined endoscopically as a distinct, visible crater >5 mm in diameter with a slough base. 

### 2.6. Statistical Analysis

The descriptive patient characteristics were summarized, and the absence rate of spindle cells or vascular formations on pathology for the 116 samples was then analyzed to elucidate the FNR rate of endoscopic biopsy. To determine the cause of FNRs, the relationships between FNR and endoscopic findings (size, site, macroscopic appearance) were evaluated using the *χ* ^2^ test. Those factors that emerged as significant (*P* < 0.10) on univariate analysis were included in a multiple exact logistic regression model. A final model was then developed by backward selection of factors showing values of *P* < 0.10 and odds ratios (ORs) and 95% confidence intervals (CIs) estimated.

Values of *P* < 0.10 were considered significant. All statistical analyses were performed using Stata version 10 software (StataCorp LP, College Station, TX, USA).

## 3. Results

### 3.1. Baseline Characteristics

Characteristics of the 24 patients are shown in Table 1. All patients were males (100%) and the HIV infection route was MSM in all cases. Median CD4^+^ count was 71 cells/mL and median HIV-RNA VL was 115,000 copies/mL. GI symptoms were noted in 8 patients (33.3%) as follows: epigastric pain (*n* = 4), nausea or vomiting (*n* = 3), hematemesis (*n* = 1), melena (*n* = 1), hematochezia (*n* = 2), and diarrhea (*n* = 2) (duplicate data). 

### 3.2. Macroscopic Appearance of GI-KS on Endoscopy

A total of 113 GI-KS lesions were from 24 HIV-infected patients (Table 2). “Patches” appearance was noted in 40 lesions (34.5%) which were located mainly in the duodenum (*n* = 8, 20.0%) and right-side colon (*n* = 9, 22.5%). Only one lesion with “Polypoid” appearance was noted in the terminal ileum. “SMT” appearance was noted in 46 lesions (39.7%) located mainly in the stomach (*n* = 18, 39.1%) and duodenum (*n* = 14, 30.4%). “SMT with ulcer” appearance was noted in 26 lesions (22.4%) located mainly in the stomach (*n* = 10, 38.5%) and duodenum (*n* = 9, 34.6%). “Bulky tumor” appearance was noted in 3 lesions only in the rectum.

### 3.3. Diagnostic Yield of GI-KS on Endoscopic Biopsy

No clinical complications of GI-KS lesions were seen. There were no significant gastrointestinal bleeds or perforations, either spontaneous or after endoscopic biopsy. Diagnostic yield of GI-KS is shown in Table 3. Among the 116 lesions, 75 (64.7%) were histologically proven by endoscopic biopsy (true-positive results), while 41 (35.3%) were negative histological results (FNR) that were confirmed to have resolved following KS therapy.

Among the GI locations, the esophagus was significantly (*P* < 0.01) associated with FNR. In regards to the size of lesions, those <10 mm in diameter were significantly associated with FNR (*P* < 0.05). As for macroscopic appearance, patches were significantly associated with FNR (*P* < 0.01), while a finding of SMT with ulceration was significantly associated with true positive results (*P* < 0.05).

On multivariate analysis, the esophageal site and a patch pattern on endoscopy were independently associated with FNR (Table 4).

## 4. Discussion

Endoscopy is clearly a valuable diagnostic method for identifying GI-KS, but it may produce FNR. Firstly, we found that FNR were yielded in 41 of the 116 lesions (35.3%) in this study. Previous studies on GI-KS patients have also reported a relatively low diagnostic yield for endoscopic biopsy [17, 23–25]. Friedman et al. [17] found a diagnostic yield of 23% with a potential false-negative rate of 77%, while Saltz et al. [24] reported a diagnostic yield of 15% and thus a potential false-negative rate of 85%. Moreover, in a study of non-AIDS patients by Kolios et al. [25], biopsies resulted in a definitive diagnosis in 5 of 26 patients (19.2%). However, it is important to keep in mind that the lesions that led to the FNRs in these studies might have included non-KS GI diseases. In the present study, we defined GI-KS as biopsy specimens negative for other GI diseases and with a positive response to KS therapy, such as shrinkage or disappearance of lesions, and we strictly adhered to these diagnostic criteria.

Various clinical factors can contribute to FNR on biopsy. Submucosal location or tumor growth is considered to account for the poor diagnostic yield of standard forceps biopsies [17, 23]. Moreover, it has been suggested that the size of biopsy forceps used was 5 mm in the upper GI tract or 8 mm in the lower GI tract [17]. We also used forceps similar in size in the present study. We hypothesized that in the analysis of endoscopic biopsy of GI-KS, FNR varies with site in the GI tract, lesion size, and macroscopic appearance. 

Secondly, with regard to the GI site, we found the esophagus to be the only site associated with FNR on both univariate and multivariate analyses. In this study, we assessed the site by dividing the GI into 7 parts. Because it is difficult to differentiate the ascending colon from the cecum and the descending colon from the sigmoid colon on endoscopy, we divided the colon into the right-side and left-side colon. No other study has divided the GI tract into small segments and investigated the diagnostic yield in such detail as in the present study. The yield of endoscopic biopsy in the upper GI tract has been estimated as 13%, which is low compared with 36% on sigmoidoscopy [17]. It is not clear if the low diagnostic rate for the esophagus contributed to the result. The most likely explanation is that lesions in the esophagus had almost a patchy appearance, but the number of lesions at this site was relatively small (*n* = 4). Further prospective studies with a larger number of patients are needed.

Thirdly, in terms of the endoscopic appearance of GI-KS, small lesions (<10 mm) and patches were found to be significantly associated with FNR on univariate analysis. Because such characteristics could be confounding factors, we performed multivariate analysis and found that patches were correlated with FNR as an independent factor. We suggested that these lesions contain only a small amount of tumor tissue, which made such biopsy specimens too small to be diagnostically useful. On the other hand, the finding of “SMT with ulceration” was significantly associated with true-positive results, and these lesions were easily diagnosed by biopsy. This result may be attributable to the ulcerous appearance of the tumor, which makes it easy to obtain samples from the submucosal layer [26, 27].

Fourth, we found that endoscopic biopsy is a safe diagnostic method for GI-KS even in the presence of an ulcerative or bulky tumor. A previous report [19] also highlighted the importance of biopsy for distinguishing protruded lesions from KS and that biopsy for GI-KS was not associated with bleeding complications, which are consistent with the present results.

Endoscopists and clinicians should become familiar with characteristic endoscopic images of GI-KS and recognize that the diagnostic yield of GI-KS varies depending on the morphological features. In addition, pathologists should carefully evaluate lesions associated with FNR. Because KS-related GI lesions indicate visceral involvement, the indications for and selection of HAART and systemic chemotherapy need to be considered [8–10]. GI-KS often starts out as small patches in the early stage, and KS should not be ruled out just because the biopsy result is negative. It was recently reported that immunohistochemistry for human herpesvirus-8 (HHV8), CD31, CD34, and D2-40 is useful for differentiating KS from other gastrointestinal tumors of similar appearance [28]. When hematoxylin and eosin staining does not show characteristic proliferating spindle cells with vascular channel formations filled with blood cells, the application of such immunohistochemical analysis may reduce the frequency of FNR. 

## 5. Conclusions

Endoscopic biopsy is essential for diagnosing GI-KS and it is a safe method. While FNR were found in 35.3% of lesions, FNRs differed according to a lesion site, size, and macroscopic appearance. On endoscopic biopsy, FNR was related with early-stage KS (small size or patches appearance) and site of esophagus, whereas SMT with ulceration is relatively easy to diagnose. Caution should be exercised when performing endoscopic biopsy of these lesions in AIDS patients and evaluating the histological features.

## Figures and Tables

**Table 1:** Baseline characteristics of GI-KS patients (*N* = 24).

Age, years (IQR)	39 (34.5–49.5)
Sex, male (%)	24 (100)
MSM	24 (100)
CD4 cell counts, cells/mL (IQR)	71 (15.5, 177.5)
HIV viral load, copies/mL (IQR)	115,000 (2,900, 145,000)
GI symptoms (%)	8 (33.3%)

IQR: interquartile range; GI: gastrointestinal; MSM: men who have sex with men.

**Table 2:** Macroscopic appearances of GI-KS on endoscopy according to the GI site (*n* = 116).

GI site	Patches (*n* = 40)	Polypoid (*n* = 1)	SMT (*n* = 46)	SMT with ulcer (*n* = 26)	Bulky tumor (*n* = 3)
Upper GI	15 (37.5%)	0	33 (71.7%)	19 (73.1%)	0
Esophagus	3 (7.5%)	0	1 (2.2%)	0	0
Stomach	4 (10.0%)	0	18 (39.1%)	10 (38.5%)	0
Duodenum	8 (20.0%)	0	14 (30.4%)	9 (34.6%)	0
Lower GI	25 (62.5%)	1 (100%)	13 (28.3%)	7 (26.9%)	3 (100%)
Ileum	5 (12.5%)	1 (100%)	0	0	0
Right-side colon	9 (22.5%)	0	6 (13.0%)	5 (19.2%)	0
Left-side colon	5 (12.5%)	0	6 (13.0%)	1 (3.9%)	0
Rectum	6 (15.0%)	0	1 (2.2%)	1 (3.9%)	3 (100%)

GI: gastrointestinal; SMT: submucosal tumor.

**Table 3:** Rate and causes of false-negative endoscopic biopsy results for GI-KS lesions on univariate analysis.

	GI-KS lesions	Lesions with true-positive results	Lesions with false-negative results	*P* value
	(*n* = 116)	(*n* = 75)	(*n* = 41)	
Site				
Upper GI tract	67 (57.8%)	39 (68.3%)	28 (52.0%)	0.09
Esophagus	4 (3.45%)	0	4 (9.76%)	**<0.01**
Stomach	32 (27.6%)	19 (25.3%)	13 (31.7%)	0.46
Duodenum	31 (26.7%)	20 (26.7%)	11 (26.8%)	0.99
Ileum	6 (5.17%)	5 (6.67%)	1 (2.44%)	0.33
Lower GI tract	49 (42.2%)	36 (48.0%)	13 (31.7%)	0.09
Right-side colon	20 (17.2%)	15 (20.0%)	5 (12.2%)	0.29
Left-side colon	12 (10.3%)	8 (10.7%)	4 (7.32%)	0.88
Rectum	11 (9.48%)	8 (10.7%)	3 (7.32%)	0.56
Size <10 mm	23 (19.8%)	10 (13.3%)	13 (31.7%)	**<0.05**
Macroscopic appearance				
Patches	40 (34.5%)	18 (24.0%)	22 (53.7%)	**<0.01**
Polypoid lesion	1 (0.86%)	1 (1.33%)	0	0.46
SMT	46 (39.7%)	31 (41.3%)	15 (36.6%)	0.62
SMT with ulcer	26 (22.4%)	22 (29.3%)	4 (9.76%)	**<0.05**
Bulky mass	3 (2.59%)	3 (4.00%)	0	0.19

GI: gastrointestinal; SMT: submucosal tumor.

**Table 4:** Factors associated with false-negative endoscopic biopsy results in GI-KS lesions on multivariate analysis (*n* = 116).

	Odds ratio	95% CI	*P* value
Esophageal site	7.26	0.82–∞	0.08
Patches on endoscopy	3.30	1.33–8.36	<0.01

GI: gastrointestinal; CI: confidential interval.
